# Risk factors of distant metastasis after surgery among different breast cancer subtypes: a hospital-based study in Indonesia

**DOI:** 10.1186/s12957-020-01893-w

**Published:** 2020-05-30

**Authors:** Sumadi Lukman Anwar, Widya Surya Avanti, Andreas Cahyo Nugroho, Lina Choridah, Ery Kus Dwianingsih, Wirsma Arif Harahap, Teguh Aryandono, Wahyu Wulaningsih

**Affiliations:** 1 Division of Surgical Oncology, Department of Surgery, Dr Sardjito Hospital/Faculty of Medicine, Public Health, and Nursing, Universitas Gadjah Mada, Jl Kesehatan No. 1, Yogyakarta, 55281 Indonesia; 2 Department of Radiology, Dr Sardjito Hospital/Faculty of Medicine, Public Health, and Nursing, Universitas Gadjah Mada, Yogyakarta, 55281 Indonesia; 3 Department of Anatomical Pathology, Dr Sardjito Hospital/Faculty of Medicine, Public Health, and Nursing, Universitas Gadjah Mada, Yogyakarta, 55281 Indonesia; 4 Division of Surgical Oncology, Department of Surgery, Dr M Jamil Hospital/Faculty of Medicine, Universitas Andalas, Padang, 25127 Indonesia; 5 MRC Unit for Lifelong Health and Ageing, University College London, Place London, Bedford 33, London, WC1B 5JU UK

**Keywords:** Metastasis, Breast cancer, Subtypes, Luminal, Triple negative

## Abstract

**Background:**

More than one third of breast cancer patients including those that are diagnosed in early stages will develop distant metastasis. Patterns of distant metastasis and the associated risks according to the molecular subtypes are not completely revealed particularly in populations of patients with delayed diagnosis and advanced stages.

**Methods:**

Breast cancer patients (*n* = 1304) admitted to our institute (2014–2017) were evaluated to identify the metastatic patterns and the associated risks. Metastatic breast cancers at diagnosis were found in 245 patients (18.7%), and 1059 patients were then grouped into non-metastatic and metastatic groups after a median follow-up of 3.8 years.

**Results:**

Infiltration of the tumor to the skin and chest wall prevailed as the most powerful predictor for distant metastasis (OR 2.115, 95% CI 1.544–2.898) particularly in the luminal A-like subtype (OR 2.685, 95% CI 1.649–4.371). Nodal involvement was also significantly associated with the risk of distant metastasis (OR 1.855, 95% CI 1.319–2.611), and the risk was higher in the Luminal A-like subtype (OR 2.572, 95% CI 1.547–4.278). Luminal A-like subtype had a significant higher risk of bone metastasis (OR 1.601, 95% CI 1.106–2.358). In respect to treatment, a combination of anthracyclines and taxanes-based chemotherapy was significantly associated with lower distant organ spread in comparison with anthracycline-based chemotherapy (OR 0.510, 95% CI 0.355–0.766) and the effect was stronger in Luminal A-like subtype (OR 0.417, 95% CI 0.226–0.769). Classification into Luminal and non-Luminal subtypes revealed significant higher risks of bone metastasis in the Luminal subtype (OR 1.793, 95% CI 1.209–2.660) and pulmonary metastasis in non-Luminal breast cancer (OR 1.445, 95% CI 1.003–2.083).

**Conclusion:**

In addition to guiding the treatment plan, a comprehensive analysis of clinicopathological variables including the molecular subtypes could assist in the determination of distant metastasis risks of breast cancer patients. Our study offers new perspectives concerning the risks of distant metastasis in breast cancer subtypes in order to plan intensive surveillance or escalation of treatment particularly in a setting where patients are predominantly diagnosed in late stages.

## Introduction

Breast cancer is ranked as the most frequently diagnosed cancer among women worldwide [1]. The increasing incidence of breast cancer is accompanied by significant decreases in the mortality rate particularly in high-income countries due to the recent implementation of early detection and multidisciplinary treatment approaches involving individualized surgery, chemotherapy, radiotherapy, hormonal therapy, and targeted therapy [2]. However, the proportion of case fatality rates in low- and middle-income countries (LMICs) including in Indonesia is significantly higher than in high-income countries [2, 3]. Several factors including demographic, socioeconomic, and healthcare system factors are associated with the higher mortality rates in LMICs [2–4]. In the clinical course of cancer, mortality is primarily caused by distant metastasis [5, 6]. Among breast cancer patients, approximately one third of them will eventually develop distant spread including those that are diagnosed in early stages [7]. Identification of metastasis-associated risks is potentially useful to improve breast cancer management.

Recent studies have revealed several intrinsic subtypes of breast cancer according to the expression profiling, i.e., luminal A, luminal B, HER-2 enriched, basal-like, and normal-like subtypes which are useful to guide more precise treatments and to possibly predict relapse and survival [8, 9]. Further studies showed that expression profiling-based subclassification could be substituted by immune-histochemical staining of estrogen receptors (ER), progesterone receptors (PR), and human epidermal growth factor receptor-2 (HER-2) [10, 11]. Epidemiological studies have shown the association of distant metastasis risks across different breast cancer subtypes [12]. For example, the mortality rate of patients with ER negative is relatively higher than those with ER positive [13, 14]. However, the available evidence of distant metastasis risk factors is mainly developed from patients in the early stages of breast cancer after receiving concomitant treatment of surgery, chemotherapy, and hormonal therapy [7, 12]. There are relatively limited studies reporting risks of distant metastasis among patients with predominantly advanced stages.

Although incidence, clinical course, and survival of breast cancer vary in different ethnic groups, socioeconomic backgrounds, and geographic locations, most studies concerning distant metastasis risks in different breast cancer subtypes are derived from European and North American countries [11, 12]. Identification of distant metastasis risk factors is potentially useful to design better surveillance programs as well as modification of treatment intensification particularly for high-risk patients. Using a cohort of breast cancer patients from an indigenous population of predominantly Javanese-Indonesians, this study evaluated risk factors for the development of distant metastasis after surgery in different breast cancer molecular subtypes defined by expression of ER, PR, and HER2.

## Materials and methods

### Study population and design

Overall, 1304 breast cancer patients were treated at the Department of Surgery, Dr. Sardjito Hospital, in 2013–2018 representing ~ 60% of all breast cancer patients diagnosed in Yogyakarta and the south part of Central Java province. There were 245 patients (18.7%) diagnosed with metastatic cancer at diagnosis, and the remaining 1059 (81.3%) patients without distant metastasis at diagnosis were then included in the analysis. Patients were recruited according to the following eligibility criteria: definitive diagnosis of breast cancer and received standard treatment, as well as follow-up at the Dr. Sardjito Hospital. The study was approved by the Medical and Health Research Ethics Committee Faculty of Medicine, Public Health, and Nursing—Universitas Gadjah Mada Yogyakarta (1143/EC/2017 and 1049/EC/2018).

### Data collection

Information of demographic data, clinical and tumor characteristics including age, cancer stages, tumor size, lymph node involvement, regional extension, and distant metastasis, histological grades, vascular and perineural infiltration, and delivered treatment (surgery, chemotherapy, radiotherapy, and hormonal therapy) were extracted from the medical records. The staging of breast cancer was determined using the tumor-node-metastasis (TNM) system following the guidelines of the 7th Edition of American Joint Committee on Cancer (AJCC) [15]. Histological type of breast cancer was classified based on the World Health Organization (WHO) guidelines [16]. Additionally, histological grade of the primary tumor was determined using the modified Scarff-Bloom and Richardson system (mSBR) [17]. Records of invasion to surrounding soft tissues including vascular, lymphatic, and neural system were extracted from the pathology reports. Expressions of ER, PR, HER2, and Ki-67 were analyzed using immunohistochemistry staining and graded as previously described [18]. ER and PR were determined as positive if staining of the nuclear tumor cells was more than 1% of total tumor cells. HER2 was considered positive if the IHC staining was 3+. HER2 2+ or ambiguous results were considered positive if fluorescence in situ hybridization (FISH) showed amplification. In the absence of FISH or CISH data, HER2 2+ was considered negative.

Intrinsic subtypes of breast cancer were classified based on the modified criteria of St. Gallen Consensus 2013 [11, 19] using receptor and proliferation markers into luminal A-like (positive expression of ER or PR, HER2^−^, and Ki67 < 20% or low grade), luminal B-like (positive expression of ER or PR, HER2^+^, and Ki67 > 20% or high grade), HER2-enriched (ER^−^/PR^−^/HER2^+^), and triple-negative (ER^−^/PR^−^/HER2^−^).

Risk factors were assessed from interviews during the diagnosis of breast cancer. Measurements of weight and height were obtained from the patients’ medical records as part of standard clinical procedures to calculate body surface area (BSA) prior to chemotherapy or drug dose calculation. Body mass index (BMI) was classified according to the World Health Organization criteria into underweight (< 18.5 kg/m^2^), normal weight (18.5–24.9 kg/m^2^), overweight (24.9–29.9 kg/m^2^), and obese (≥ 30 kg/m^2^). Menarche was classified into early menarche (≤ 12 years), normo-menarche (13–14 years), and late menarche (≥ 15 years). Menopause was classified according to the age at which the period ceased (≤ 50/> 50 years old). Breastfeeding was determined as yes, if performed for at least 1 year, or no, if less than 1 year. The residence of patients was classified into urban (*kota*) and rural (*desa*) according to the patients’ address shown in their identity card at the time of diagnosis and formal governmental administrative status of the residence. Parity was grouped into null- or multi-parity according to the history of full-term pregnancies. Education levels were determined according to the government education system of elementary (6 years), junior and high school (3 years each), and graduate school.

### Follow-up

The main outcome of the study was evidence of distant metastasis, defined as the presence of cancer spread to the lung, bone, liver, and brain indicated with clinical manifestations and confirmed with imaging/pathology examination and/or radiologic changes confirmed with computed tomography imaging with contrast or whole-body bone scan. Surveillance of the patients was performed according to the institutional guidelines. Follow-up visits after acute treatment of surgery, chemotherapy, and radiotherapy were scheduled at least once a month in the first 6 months and then every 6 months afterward unless any unscheduled admission was indicated. Comprehensive examinations including a thorough clinical examination, breast sonography and/or mammography, abdominal ultrasonography, chest X-ray, and bone scan were performed following the national recommendations. Any documented cancer progression, cancer-related mortality, and significant clinical findings were recorded until the last date of the follow-up study in July 2019.

### Statistical analysis

Frequency tables were presented to compare attributable clinicopathological risk factors of distant metastasis across different breast cancer subtypes. Continuous variables were presented in means or medians ± standard deviation (SD) or standard error (SE). Categorical variables were compared using the *χ*^2^ tests, and continuous variables were compared using the Mann-Whitney *U* tests. The association was then analyzed using multivariable logistic regression with distant metastasis during follow-up as the dependent variable and clinicopathological determinants as covariates overall and stratified by breast cancer subtype. We additionally assessed the advanced stage at diagnosis as the dependent variable with sociodemographic determinants as covariates. We also performed univariable and multivariable logistic regression analyses of overall and site-specific distant metastasis by breast cancer subtype. When analyzing each metastasis site, the remaining patients without distant metastasis to a given site were used as the controls. All statistical analyses were performed using SPSS 17.0 software (SPSS Inc., Chicago). All comparisons were two-sided, and *P* < 0.05 was used as the cut-off for a statistically significant difference.

## Results

### Baseline characteristics of participants

We presented baseline characteristics for the main population of 1304 breast cancer patients, who had a median age at diagnosis of 51 years old and a mean BMI of 24 kg/m^2^. The majority of patients were of Javanese ethnicity (*n* = 1273, 97.6%), lived in the rural area (*n* = 979, 75.1%), diagnosed in the late stages (III–IV, *n* = 952, 73%), and had hormonal receptor-positive tumors (*n* = 751, 57.6%) (Table 1). The median age for menarche was 14 years old with 17.3% of women reported having menarche at the age of less than 12 years old. The majority of patients were diagnosed after menopause (*n* = 941, 72%), and 24% of them reported having menopause after age 50. Most patients had primary tumors larger than 5 cm (*n* = 902, 69.2%) and positive axillary lymph node (N1–3, *n* = 996, 76.4%) at diagnosis. The majority of tumors were luminal A-like subtype (45.4%, *n* = 592) followed by triple-negative breast cancer (TNBC) (26.3%, *n* = 343), Her2-enriched (16.1%, *n* = 210), and luminal B-like (12.2%, *n* = 159). Distributions of baseline age, ethnicity, age at menarche, age at menopause, parity, breastfeeding practice, family history, BMI, histological grades, tumor size, and axillary lymph node infiltration were not significantly different among intrinsic breast cancer subtypes (Table 1).

**Table 1:** Characteristics of study participants. Distribution of demographic and clinicopathological variables of overall breast cancer patients and the characteristics by tumor subtypes

Variables	Category	Overall	Luminal (*N* = 750)		Non-luminal (*N* = 554)		*P* value
			Luminal A-like	Luminal B-like	Her2-enriched	TNBC	
Age (year old)		1304 (100%)	592 (45.4%)	159 (12.2%)	210 (16.1%)	343 (26.3%)	
	Mean (range)	50.9 (23.90)	51.5 (27.90)	49 (30.72)	51 (24.78)	50.6 (23.83)	
	≤ 35	85 (6.5%)	33 (5.6%)	12 (7.5%)	15 (7.2%)	25 (7.3%)	0.059
	36–40	148 (11.4%)	49 (8.3%)	24 (15.1%)	22 (10.1%)	53 (15.5%)	
	41–55	637 (48.8%)	312 (52.7%)	75 (47.2%)	103 (49.8%)	147 (42.9%)	
	56–65	333 (25.6%)	150 (25.3%)	39 (24.5%)	56 (26.6%)	88 (25.6%)	
	> 65	101 (7.7%)	48 (8.1%)	9 (5.7%)	14 (6.3%)	30 (8.7%)	
Ethnicity	Javanese	1273 (97.6%)	578 (97.6%)	156 (98.1%)	206 (98.1%)	333 (97.1%)	0.808
	Non-Javanese	31 (2.4%)	14 (2.4%)	3 (1.9%)	4 (1.9%)	10 (2.9%)	
Menarche (year old)	≤ 12	225 (17.3%)	90 (15.2%)	25 (15.7%)	39 (18.6%)	71 (20.7%)	0.369
	13–14	605 (46.4%)	275 (46.5%)	77 (48.4%)	94 (44.8%)	159 (46.4%)	
	≥ 15	474 (36.3%)	227 (38.3%)	57 (38.5%)	77 (36.7%)	113 (32.9%)	
Menopause age (year old)	≤ 50	715 (76%)	356 (79.6%)	75 (70%)	114 (72.2%)	170 (73.6%)	**0.009**
	> 50	226 (24%)	91 (20.4%)	31 (30%)	43 (27.8%)	61 (26.4%)	
Parity	Nulliparous	138 (10.6%)	62 (10.5%)	23 (14.5%)	17 (8.2%)	36 (10.5%)	0.288
	Multiparous	1166 (89.4%)	530 (89.5%)	136 (85.5%)	193 (91.8%)	307 (89.5%)	
Breastfeeding	No	252 (19.3%)	120 (20.3%)	34 (21.4%)	34 (16.4%)	64 (18.7%)	0.576
	Yes	1052 (80.7%)	472 (79.7%)	125 (78.6%)	176 (83.6%)	279 (81.3%)	
BMI	≤ 18.5	172 (13.2%)	80 (13.5%)	23 (14.5%)	28 (13.3%)	41 (12.0%)	0.956
	18.5–25	661 (50.7%)	298 (48.3%)	76 (47.8%)	109 (51.9%)	178 (51.9%)	
	25–30	348 (26.7%)	158 (27.9%)	48 (30.2%)	54 (25.7%)	88 (25.7%)	
	> 30	123 (9.4%)	56 (10.3%)	12 (7.5%)	19 (9.0%)	36 (10.5%)	
Family history	Yes	234 (17.9%)	98 (16.6%)	27 (17%)	44 (21.0%)	65 (19%)	0.449
	No	1070 (82.1%)	494 (83.4%)	132 (83%)	166 (79.0%)	278 (81%)	
Histology grade	I	8 (0.7%)	3 (0.5%)	2 (1.3%)	0 (0%)	3 (0.9%)	0.411
	II	247 (18.9%)	122 (20.6%)	29 (18.2%)	38 (18.1%)	58 (16.9%)	
	III	1049 (80.4%)	467 (78.9%)	128 (80.5%)	172 (81.9%)	282 (82.2%)	
Stage	I	11 (0.9%)	6 (1%)	1 (0.6%)	2 (1%)	2 (0.6%)	**0.005**
	II	343 (26.3%)	187 (31.6%)	31 (19.5%)	50 (23.8%)	75 (21.9%)	
	III	705 (54.2%)	292 (49.3%)	90 (56.6%)	118 (56.2%)	205 (59.8%)	
	IV	245 (18.8%)	107 (18.1%)	37 (23.3%)	40 (19.0%)	61 (17.8%)	
Tumor size	≤ 2 cm	57 (4.3%)	31 (5.3%)	7 (4.4%)	10 (4.8%)	9 (2.6%)	0.077
	2–5 cm	345 (26.5%)	169 (28.5%)	39 (24.5%)	58 (27.6%)	79 (23%)	
	> 5 cm	902 (69.2%)	392 (66.2%)	113 (71.1%)	142 (67.6%)	255 (74.4%)	
Node status	N0	308 (23.6%)	161 (27.2%)	28 (17.6%)	46 (21.9%)	73 (21.3%)	0.054
	N1	687 (52.7%)	312 (52.7%)	84 (52.8%)	106 (50.5%)	185 (53.9%)	
	N2	250 (19.2%)	92 (15.5%)	38 (23.9%)	47 (22.4%)	73 (21.3%)	
	N3	59 (4.5%)	27 (4.6%	9 (5.7%)	11 (5.2%)	12 (3.5%)	
Endocrine therapy	No	555 (42.6%)	11 (1.9%)	1 (0.6%)	206 (98.1%)	337 (98.3%)	0.118
	Tamoxifen	389 (29.8%)	294 (49.7%)	87 (54.7%)	2 (1%)	6 (1.7%)	
	Aromatase inhibitor	360 (27.6%)	287 (48.5%)	71 (44.7%)	2 (1%)	0 (0%)	
Chemotherapy	No	203 (15.6%)	91 (15.4%)	21 (13.2%)	35 (16.7%)	56 (16.3%)	0.772
	Yes	1101 (84.6%)	501 (84.6%)	138 (86.8%)	175 (83.3%)	287 (83.7%)	
Radiotherapy	No	490 (37.6%)	225 (38%)	60 (37.7%)	89 (42.4%)	116 (33.8%)	0.118
	Yes	814 (62.4%)	367 (62%)	99 (62.3%)	121 (57.6%)	227 (66.2%)	
Surgery	Mastectomy	1069 (82%)	473 (79%)	124 (78%)	180 (85.7%)	292 (85.1%)	0.172
	BCT	99 (7.6%)	49 (8.3%)	13 (8.2%)	14 (6.7%)	23 (6.7%)	
	Biopsy	136 (10.5%)	70 (11.8%)	22 (13.8%)	16 (7.6%)	28 (8.2%)	

Metastatic breast cancer at diagnosis was found in 245 (18.7%) patients. After a median follow-up of 3.8 years, the distant spread was further detected in 271 patients (25.6%) from the remaining cohort (*n* = 1059) and included in subsequent analyses.

### Associations of sociodemographic and clinicopathological factors with advanced stage at diagnosis

We presented the correlation of sociodemographic variables with advanced breast cancer stages at diagnosis in Table 2. Education lower than high school was significantly associated with late-stage breast cancer diagnosis (OR 2.288, 95% CI 1.740–3.007), with *P* < 0.0001. In addition, residence in a rural area was also significantly correlated with an advanced stage of breast cancer diagnosis (OR 5.558, 95% CI 4.171–7.046), with *P* < 0.0001. The multivariable regression analysis showed that the variables were significantly associated with a breast cancer diagnosis at an advanced stage, *F*(11, 1191) = 157.9, with *P* < 0.0001, and *R*^*2*^ = 0.192. Residence in a rural area and lower education were significantly correlated to advanced stage at diagnosis with 62% and 42.7% efficiency, respectively.

**Table 2:** Odds ratios and confidence intervals for advanced stages at diagnosis in 1059 breast cancer patients

Variable	Category	Stage		Univariable analysis		Multivariable analysis	
				OR (95% CI)	*P* value	OR (95% CI)	*P* value
		Advance (*n*)	Early (*n*)				
Age	≤ 40 years old	116	69	1.230 (0.884–1.709)	0.220	0.868 (0.544–1.384)	0.551
	> 40 years old	589	285	Ref		Ref	
Breastfeeding	Yes	566	281	0.945 (0.688-1.298)	0.745	0.897 (0.556-1.445)	0.654
	No	139	73	Ref		Ref	
Marital status	Not married	43	19	Ref		Ref	
	Married	662	335	0.873 (0.501–1.522)	0.625	0.858 (0.473–1.557)	0.614
Education	Lower than high school	339	102	2.288 (1.741–3.008)	**< 0.0001**	1.533 (1.132–2.075)	**0.006**
	High school and graduate	366	252	Ref		Ref	
Parity	Nulliparity	75	42	0.884 (0.592–1.321)	0.559	0.923 (0.505–1.668)	0.779
	Multiparity	630	312	Ref		Ref	
Ethnicity	Javanese	690	347	0.928 (0.375–2.297)	0.998	0.506 (0.190–1.350)	0.174
	Non-Javanese	15	7	Ref		Ref	
BMI	≤ 25	450	212	1.182 (0.909–1.536)	0.211	1.060 (0.794–1.416)	0.693
	> 25	255	142	Ref		Ref	Ref
Menarche	≤ 14 years old	456	237	Ref		Ref	Ref
	> 14 years old	249	117	1.106 (0.844–1.449)	0.464	1.058 (0.788–1.420)	0.631
Menopause	≤ 50 years old	397	185	1.164 (0.820–1.653)	0.396	1.120 (0.855–1.468)	0.409
	> 50 years old	118	64	Ref		Ref	Ref
Family history	Yes	126	70	0.883 (0.638–1.222)	0.465	0.877 (0.623–1.246)	0.508
	No	579	284	Ref		Ref	
Residence	Rural	586	166	5.577 (4.185–7.431)	**< 0.0001**	5.096 (3.765–6.896)	**< 0.0001**
	Urban	119	188	Ref		Ref	

Association of sociodemographic variables with advance stages at diagnosis were analyzed using univariable and multivariable binary logistic regression

*Ref* reference, *OR* odd ratio, *CI* confidence interval, *BMI* body mass index

### Associations of sociodemographic and clinicopathological factors with distant metastasis during follow-up

With regard to distant metastasis, having menopause older than 50 years old and younger than 40 years old at diagnosis were significantly correlated with higher risks of distant metastasis (OR 1.577, 95% CI 1.121–2.137, and OR 1.548, 95% CI 1.121–2.137), with *P* = 0.008, respectively, as shown in Tables 3 and 4. Additionally, living in a rural area was also associated with a higher risk of distant metastasis (OR 1.548, 95% CI 1.121–2.137), with *P* = 0.008.

**Table 3:** Odds ratios and 95% confidence intervals of distant metastasis across different breast cancer subtypes. Association of clinicopathological variables with distant metastasis was analyzed using univariable binary logistic regression

Variables	Category	Overall (*N* = 1059)			Luminal A (*N* = .485)			Luminal B (*N* = 122)			Her2-enriched (*N* = 170)			TNBC (*N* = 282)		
					Luminal (*N* = 607)						Non-luminal (*N* = 452)					
		OR	95% CI	*P* value	OR	95% CI	*P* value	OR	95% CI	*P* value	OR	95% CI	*P* value	OR	95% CI	*P* value
Age (years)	≤ 40	1.773	1.262–2.493	**0.001**	2.283	1.321–3.953	**0.003**	1.255	0.440–3.584	0.670	2.037	0.901-4.608	0.087	1.333	0.730–2.433	0.348
	> 40	Ref	Ref		Ref	Ref		Ref	Ref		Ref	Ref		Ref	Ref	
Menarche	≤ 12	0.946	0.656–1.364	0.767	0.998	0.567–1.758	0.995	2.571	0.931–7.092	0.068	0.824	0.327–2.075	0.681	0.620	0.309–1.245	0.180
	> 12	Ref	Ref		Ref	Ref		Ref	Ref		Ref	Ref		Ref	Ref	
Menopause	≤ 50	0.626	0.409–0.957	**0.030**	0.792	0.423–1.482	0.466	0.939	0.317–2.783	0.317	0.451	0.143–1.426	0.175	0.401	0.166–0.967	0.042
	> 50	Ref	Ref		Ref	Ref		Ref	Ref		Ref	Ref		Ref	Ref	
Parity	Nulliparity	1.301	0.817–2.071	0.268	1.049	0.553–1.993	0.883	5.385	0.680–42.66	0.111	2.069	0.444–9.646	0.269	1.037	0.441–2.439	0.934
	Multiparity	Ref	Ref		Ref	Ref		Ref	Ref		Ref	Ref		Ref	Ref	
Breastfeeding	Yes	Ref	Ref		Ref	Ref		Ref	Ref		Ref	Ref		Ref	Ref	
	No	1.108	0.780–1.572	0.567	1.170	0.702–1.950	0.546	2.685	0.741–9.729	0.149	1.095	0.433–2.772	0.848	1.329	0.696–2.544	0.388
BMI	≥ 25	0.832	0.623–1.110	0.603	1.064	0.699–1.619	0.774	0.913	0.381–2.189	0.935	0.415	0.188–0.915	**0.029**	0.782	0.449–1.362	0.385
	< 25	Ref	Ref		Ref	Ref		Ref	Ref		Ref	Ref		Ref	Ref	
Family history	Yes	1.061	0.747–1.511	0.785	1.153	0.659–2.018	0.659	2.342	0.859–6.410	0.096	1.169	0.511–2.674	0.711	0.968	0.500–1.873	0.922
	No	Ref	Ref		Ref	Ref		Ref	Ref		Ref	Ref		Ref	Ref	
Grade	I–II	Ref	Ref		Ref	Ref		Ref	Ref		Ref	Ref		Ref	Ref	
	III	1.174	0.822–1.678	0.378	0.993	0.605–1.627	0.976	1.398	0.430–4.546	0.578	1.708	0.607–4.802	0.310	1.273	0.627–2.583	0.504
Stage	I–II	Ref	Ref		Ref	Ref		Ref	Ref		Ref	Ref		Ref	Ref	
	III	1.883	1.373–2.646	**< 0.0001**	2.160	1.377–3.389	**0.001**	1.156	0.439–3.039	0769	2.227	0.949–5.208	0.066	1.616	0.863–3.021	0.134
Tumor size	≤ 5	Ref	Ref		Ref	Ref		Ref	Ref		Ref	Ref		Ref	Ref	
	> 5	1.115	0.829–1.501	0.472	0.886	0.582–1350	0.969	2.068	0.716–5.971	0.179	1.985	0.897–4.389	0.091	0.978	0.543–1.762	0.940
T	T1–2	Ref	Ref		Ref	Ref		Ref	Ref		Ref	Ref		Ref	Ref	
	T3–4	1.429	1.015–2.012	**0.041**	1.162	0.732–1.845	0.525	1.605	0.498–5.169	0.428	2.440	0.948–6.276	0.064	1.624	0.768–3.345	0.204
	T1–3	Ref	Ref		Ref	Ref		Ref	Ref		Ref	Ref		Ref	Ref	
	T4	2.115	1.544–2.898	**< 0.0001**	2.685	1.649–4.371	**< 0.0001**	2.380	0.964–5.876	0.060	1.569	0.708–3.478	0.267	1.709	0.957–3.054	0.070
N	N0	Ref	Ref		Ref	Ref		Ref	Ref		Ref	Ref		Ref	Ref	
	N1–3	1.855	1.319–2.611	**< 0.0001**	2.571	1.548–4.274	**< 0.0001**	0.807	0.300–2169	0.671	1.736	0.704–4.292	0231	1.515	0.797–2.882	0.205
Chemotherapy	Anthracycline	Ref	Ref		Ref	Ref		Ref	Ref		Ref	Ref		Ref	Ref	
	Anthracycline-taxane	0.510	0.355–0.766	**0.001**	0.417	0.226–0.769	**0.005**	0.629	0.228–1.734	0.370	0.769	0.329–1.798	0.544	0.518	0.237–1.131	0.099
Radiotherapy	Yes	Ref	Ref		Ref	Ref		Ref	Ref		Ref	Ref		Ref	Ref	
	No	1.788	1.269–2.521	**0.001**	1.923	1.160–3.187	**0.011**	0.861	0.322–2.303	0.766	2.304	0.984–5.398	0.055	1.794	0.898–3.585	0.098
Surgery	MRM	Ref	Ref		Ref	Ref		Ref	Ref		Ref	Ref		Ref	Ref	
	BCT	1.005	0.624–1.618	0.995	0.837	0.414–1.694	0.621	0.996	0.254–3902	0.995	1.242	0.368–4.190	0727	1.175	0.464–2.977	0.734

*Ref* reference, *OR* odd ratio, *CI* confidence interval, *TNBC* triple negative breast cancer, *BMI* body mass index, *T* primary tumor size, *N* involved lymph nodes

**Table 4:** Odds ratios and 95% confidence intervals of distant metastasis across different breast cancer subtype. Association of clinicopathological variables with distant metastasis were analyzed using multivariable binary logistic regression

Variables	Category	Overall (*N* = 1059)			Luminal A (*N* = .485)			Luminal B (*N* = 122)			Her2-enriched (*N* = 170)			TNBC (*N* = 282)		
					Luminal (*N* = 607)						Non-luminal (*N* = 452)					
		OR	95% CI	*P* value	OR	95% CI	*P* value	OR	95% CI	*P* value	OR	95% CI	*P* value	OR	95% CI	P value
Age (years)	≤ 40	1.414	0.892–2.242	0.140	2.118	1.028–4.367	**0.042**	1.112	0.235–5.347	0.885	1.278	0.229–3.030	0.782	1.340	0.565–3.174	0.507
	> 40	Ref	Ref		Ref	Ref		Ref	Ref		Ref	Ref		Ref	Ref	
Menarche	≤ 12	1.099	0.753–1.605	0.624	1.059	0.583–1.924	0.850	2.222	0.645–7.633	0.205	1.005	0.363–2.782	0.993	1.857	0.893–3.862	0.098
	> 12	Ref	Ref		Ref	Ref		Ref	Ref		Ref	Ref		Ref	Ref	
Menopause	≤ 50	0.814	0.614–1.078	0.151	0.902	0.590–1.378	0.466	1.098	0.441–2.734	0.840	0.396	0.170–0.923	**0.032**	0.997	0.578–1.720	0.991
	> 50	Ref	Ref		Ref	Ref		Ref	Ref		Ref	Ref		Ref	Ref	
Parity	Nulliparity	1.329	0.699–2.527	0.385	0.685	0.259–1.815	0.447	3.541	0.262–47.87	0.111	4.100	0.545–30.823	0.170	1.989	0.588–6.724	0.268
	Multiparity	Ref	Ref		Ref	Ref		Ref	Ref		Ref	Ref		Ref	Ref	
Breastfeeding	Yes	Ref	Ref		Ref	Ref		Ref	Ref		Ref	Ref		Ref	Ref	
	No	0.968	0.595–1.573	0.895	1.612	0.740–3.509	0.229	1.614	0.327–7.973	0.557	0.607	0.167–2.209	0.449	0.526	0.210–1.317	0.170
BMI	≥ 25	0.853	0.632–1.153	0.301	1.061	0.679–1.659	0.794	0.872	0.306–2.487	0.557	0.408	0.175–0.951	**0.038**	0.857	0.475–1.547	0.609
	< 25	Ref	Ref		Ref	Ref		Ref	Ref		Ref	Ref		Ref	Ref	
Family history	Yes	0.944	0.656–1.395	0.785	1.126	0.624–2.030	0.694	3.514	0.933–10.638	0.065	0.842	0.323–2.193	0.724	1.046	0.526–1.317	0.170
	No	Ref	Ref		Ref	Ref		Ref	Ref		Ref	Ref		Ref	Ref	
Grade	I–II	Ref	Ref		Ref	Ref		Ref	Ref		Ref	Ref		Ref	Ref	
	III	1.096	0.754–1.594	0.631	0.986	0.573–1.697	0.961	1.578	0.396–6.296	0.578	1.545	0.488–4.892	0.459	1.182	0.562–2.486	0.660
Stage	I–II	Ref	Ref		Ref	Ref		Ref	Ref		Ref	Ref		Ref	Ref	
	III	1.220	0.702–2.119	0.481	1.284	0.579–2.072	0.569	1.244	0.161–9.635	0.769	0.837	0.165–4.248	0.830	0.917	0.282–2.982	0.885
Tumor size	≤ 5	Ref	Ref		Ref	Ref		Ref	Ref		Ref	Ref		Ref	Ref	
	> 5	0.938	0.664–1.325	0.938	0.719	0.436–1.186	0.719	2.372	0.664–8.474	0179	1.778	0.635–4.977	0.273	0.876	0.443–1.734	0.885
T	T1–3	Ref	Ref		Ref	Ref		Ref	Ref		Ref	Ref		Ref	Ref	
	T4	1.887	1.327–2.682	**< 0.0001**	2.485	1.432–4.313	**0.001**	2.550	0.814–7.991	0.108	1.778	0.635–4.977	0.273	1.642	0.846–3.189	0.143
N	N0	Ref	Ref		Ref	Ref		Ref	Ref		Ref	Ref		Ref	Ref	
	N1–3	1.402	0.870–2.262	0.165	2.000	1.028–3.891	**0.041**	1.380	0.211–9.034	0.737	0.912	0.235–3.543	0.894	0.792	0.279–2.252	0.662
Chemotherapy	Anthracycline	Ref	Ref		Ref	Ref		Ref	Ref		Ref	Ref		Ref	Ref	
	Anthracycline-taxane	0.785	0.656–0.941	**0.009**	0.680	0.426–0.968	**0.045**	0.570	0.241–1.347	0.200	0.859	0.539–1.369	0.523	0.724	0.505–1.037	0.078
Radiotherapy	Yes	Ref	Ref		Ref	Ref		Ref	Ref		Ref	Ref		Ref	Ref	
	No	1.244	0.815–1.897	0.312	0.869	0.452–1.672	0.674	1.515	0.385–5.694	0.553	1.428	0.338–6.036	0.055	0.643	0.279–1.483	0.300
Surgery	MRM	Ref	Ref		Ref	Ref		Ref	Ref		Ref	Ref		Ref	Ref	
	BCT	1.203	0.751–1.927	0.443	0.913	0.459–1.817	0.796	1.420	0.296–6.815	0.662	1.242	0.368–4.190	0.727	1.215	0.424–3.479	0.717

*Ref* reference, *OR* odd ratio, *CI* confidence interval, *TNBC* triple negative breast cancer, *BMI* body mass index, *T* primary tumor size, *N* involved lymph nodes

Although larger tumor did not directly correlate with distant spread, tumor larger than 5 cm or with infiltration to the skin or chest wall (T3–4) was significantly associated with distant metastasis (OR 1.429, 95% CI 1.015–2.012), with *P* = 0.041. Infiltration to the skin or chest wall (T4) was the most robust predictor for distant metastasis (OR 2.605, 95% CI 1.505–2.835), with *P* < 0.001. Distant metastasis rate was higher in histologically high grade in comparison with low and moderate grade (26% vs 23%) although the association was not statistically significant (OR 1.164, 95% CI 0.814–1.664), with *P* = 0.405 (Tables 3 and 4).

Surgery type was not significantly different in patients who later developed distant spread or without metastasis (89.6% vs 90.1% for mastectomy and 9.3% vs 9.4% for breast conservation surgery). In respect to the clinical stages, the extent of surgery types did not correlate with the risk of metastasis (Tables 3 and 4).

In total, 84.6% patients received chemotherapy and the patients were then stratified according to the regimens: 70.3% received anthracyclines-based chemotherapy (*n* = 745), 20.8% received anthracycline and taxanes-based chemotherapy (*n* = 220), 0.3% received schedule without anthracyclines (*n* = 2), and 8.7% did not receive chemotherapy (*n* = 92). Administration of the combination of anthracyclines and taxanes-based chemotherapy was associated with lower distant metastasis risk (OR 0.510, 95% CI 0.355–0.766), with *P* = 0.001 particularly in luminal A-like subtype (OR 0.417, 95% CI 0.226–0.769), with *P* = 0.005, as shown in Tables 3 and 4.

More than 60% of patients received radiotherapy. In respect to the intrinsic breast cancer subtypes, there was no significant difference in the delivery of radiotherapy. Not receiving radiotherapy was associated with higher distant metastasis (OR 1.788, 95% CI 1.269–2.521), with *P* = 0.001 (Table 3). In stage III breast cancer, no radiotherapy was associated with an increased risk of distant spread (OR 2.059, 95% CI 1.079–3.927), with *P* = 0.028.

A multivariable regression analysis of clinicopathological variables showed significant association with the risk of distant metastasis (Table 4), *F*(15, 1148) = 56.23, with *P* < 0.0001, and *R*^2^ = 0.076. Tumor infiltration to skin and chest wall and combination of chemotherapy were significantly correlated to the risk of distant metastasis with 63.5%, and 24.1% efficiency, respectively.

### Stratification analysis of distant metastasis by breast cancer subtype

The majority of patients had tumor size larger than 5 cm (69.2%), and this varied according to subtypes in which TNBC had a higher proportion of larger tumors (74.4%). More than 80% of tumors were histologically graded III. Infiltration to the axillary lymph node of the axilla (ALN) at diagnosis was significantly higher in breast cancer patients who later developed metastasis (81.8% vs 69.9%). Metastasis into the regional axillary lymph nodes was higher in the non-luminal subtypes compared to the luminal subtype (75.6% vs 70.6%). Association between clinicopathological variables with risk of distant metastasis varied by intrinsic subtypes of breast cancer. For instance, the risk associated with T4 was significantly higher in the luminal A-like subtype (OR 2.685, 95% CI 1.649–4.371), with *P* < 0.0001 (Tables 3 and 4).

The positive axillary lymph node was significantly associated with a higher risk of developing metastasis (OR 1.855, 95% CI 1.319–2.611), with *P* < 0.0001 and the association was higher in luminal A-like subtype (OR 2.571, 95% CI 1.548–4.274), with *P* < 0.0001 (Tables 3 and 4).

### Association between breast cancer subtypes and distant metastasis

Among different intrinsic subtypes, TNBC had the highest rates of distant metastasis (27.3%). Non-luminal subtype had higher distant metastatic rates than the luminal subtype (26.3% vs 25.0%). ER-positive tumor had a lower frequency of distant metastasis than ER-negative tumor (25.2% vs 26.1%). Distant metastasis was lower in Her-2-positive tumor compared with Her-2-negative tumor (24.2% vs 26.1%). Using a univariate binary logistic regression, we did not find a direct association of distant metastasis risk in any specific breast cancer subtype (Table 5). A multivariable regression analysis with adjustment of age, stage, tumor size, and nodal status also did not show a significant association between particular breast cancer subtypes with the overall risk of distant metastasis.

**Table 5:** Odds ratios and 95% confidence intervals showing the association of distant metastasis risk among different breast cancer subtypes using binominal logistic regression

Molecular subtype	Metastasis	No metastasis	OR	95% CI	*P* value	Reference
Luminal A-like	123 (25.4%)	362 (74.6%)	0.973	0.737–1.283	0.849	Non-luminal A-like
Luminal B-like	29 (23.8%)	93 (76.2%)	0.892	0.574–1.389	0.615	Non-luminal B-like
Her2-enriched	42 (24.7%)	128 (75.3%)	0.946	0.647–1.383	0.773	Non-Her2-enriched
TNBC	77 (27.3%)	205 (72.7%)	1.125	0.826–1.531	0.454	Non-TNBC
Luminal	152 (25.0%)	455 (75.0%)	0.935	0.708–1.235	0.635	Non-luminal
ER positive	149 (25.2)	443 (74.8%)	0.951	0.721–1.255	0.724	ER negative
Her-2 positive	70 (24.2%)	2^9 (75.8%)	0.905	0.661–1.238	0.532	Her2 negative

*OR* odd ratio, *CI* confidence interval, *TNBC* triple negative breast cancer, *ER* estrogen receptor

Among 435 events of distant spread in 271 patients, the lung was the most common site (12.7%), followed by the bone (12.3%), pleura (8.8%), liver (5.5%), and brain (1.9%) (Table 3 and 4). Our cohort showed that 71% (*n* = 191) of patients had single-site metastasis and 29% (*n* = 78) had multiple metastatic sites.

The rates of pulmonary metastasis according to the intrinsic breast cancer subtypes were 11.9%, 7.3%, 15.3%, and 15.3% in lumina A-like, luminal B-like, Her2-enriched, and TNBC, respectively. Frequency of lung metastasis in luminal and non-luminal subtypes were 10.7% (*n* = 65) and 14.8% (*n* = 67), respectively. Non-luminal subtypes had a significant higher risk of pulmonary metastasis than the luminal subtypes (OR 1.445, 95% CI 1.003–2.083), with *P* = 0.048 as shown in Table 6.

**Table 6:** Odds ratios and 95% confidence intervals of site-specific distant metastasis by breast cancer subtype

Organ site metastasis	Breast cancer subtype	OR	95% CI	*P* value	Reference
Pulmonal metastasis					
	Molecular subtype				
	Luminal A-like	0.886	0.615–1.277	0.518	Non-luminal A-like
	Luminal B-like	0.516	0.255–1.044	0.066	Non-luminal B-like
	Her2-enriched	1.306	0.821–2.076	0.259	Non-Her2-enriched
	TNBC	1.248	0.840–1.853	0.273	Non-TNBC
	Luminal	0.692	0.480–0.997	**0.048**	Non-luminal
	Her2 positive	0.893	0.590–1.353	0.594	Her2-negative
	Axillary node positive	1.938	1.210–3.150	**0.006**	Axillary node negative
Pleural metastasis					
	Luminal A-like	0.969	0.632–1.485	0.883	Non-luminal A-like
	Luminal B-like	0.807	0.395–1.649	0.556	Non-luminal B-Like
	Her2-enriched	0.758	0.404–1.422	0.388	Non-Her2-enriched
	TNBC	1.345	0.851–2.127	0.204	Non-TNBC
	Luminal	0.895	0.584–1.374	0.613	Non-luminal
	Her2 positive	0.709	0.424–1.188	0.192	Her2-negatuve
	Axillary node positive	1.886	1.081–3.290	**0.025**	Axillary node negative
Bone metastasis					
	Luminal A-like	1.601	1.106–2.358	**0.013**	Non-luminal A-like
	Luminal B-like	1.178	0.680–1.584	2.039	Non-luminal B-Like
	Her2-enriched	0.706	0.407–1.226	0.216	Non-Her2-enriched
	TNBC	0.587	0.368–0.935	**0.025**	Non-TNBC
	Luminal	1.793	1.209–2.660	**0.005**	Non-luminal
	Her2 positive	0.894	0.603–1.362	0.603	Her2-negative
	Axillary node positive	2.093	1.285–3.411	**0.003**	Axillary node negative
Liver metastasis					
	Luminal A-like	1.231	0.722–2.100	0.446	Non-luminal A-like
	Luminal B-like	1.086	0.482–2.452	0.843	Non-luminal B-Like
	Her2-enriched	1.117	0.553–2.257	0.758	Non-Her2-enriched
	TNBC	0.645	0.329–1.263	0.201	Non-TNBC
	Luminal	1.332	0.769–2.309	0.307	Non-luminal
	Her2 positive	1.212	0.683–2.150	0.511	Her2-negative
	Axillary node positive	1.185	0.639–2.196	0.639	Axillary node negative
Brain metastasis					
	Luminal A-like	1.179	0.487–2.857	0.715	Non-luminal A-like
	Luminal B-like	0.855	0.196–3.732	0.835	Non-luminal B-Like
	Her2-enriched	0.575	0.132–2.500	0.750	Non-Her2-enriched
	TNBC	1.184	0.451–3.113	0.731	Non-TNBC
	Luminal	1.119	0.454–2.761	0.807	Non-luminal
	Her2 positive	0.661	0.219–1.996	0.501	Her2-negative
	Axillary node positive	1.123	0.404–3.118	0.824	Axillary node negative

*OR* odd ratio, *CI* confidence interval, *TNBC* triple negative breast cancer, *ER* estrogen receptor

Distant spread into the pleura was detected in 8.8% (*n* = 93) of all breast cancer patients. Frequency of pleural metastasis was higher in TNBC (10.6%, *n* = 30) in comparison with luminal A-like (8.6%, *n* = 42), luminal B-like (7.3%, *n* = 9), and Her2-enriched (7.1%, *n* = 12) although the binary logistic regression did not show significant risk (OR 1.345, 95% CI 0.851–2.127), with *P* = 0.204 as shown in Table 6. Non-luminal subtypes also showed higher frequency of pleural metastasis (9.2%, *n* = 42) compared to luminal subtypes (8.4%, *n* = 51) although the difference was not significant (OR 1.117, 95% CI 0.727–1.712), with *P* = 0.613.

The bone was the second most common site of distant spread in our cohort. Distribution across different intrinsic subtypes of breast cancer was 15.05%, 13.9%, 9.4%, and 8.5% in luminal A-like, luminal B-like, Her2-enriched, and TNBC subtype, respectively. Evaluating the associated bone metastatic risk for each breast cancer subtypes using binary logistic regression analysis, we found that luminal A-like subtypes had significantly elevated risk (OR 1.601, 95% CI 1.106–2.358), with *P* = 0.013, and TNBC had a significantly lower risk (OR 0.587, 95% CI 0.368–0.935), with *P* = 0.025. Differentiation subtypes into luminal and non-luminal also showed a higher risk of bone metastasis in luminal subtypes (OR 1.793, 95% CI 1.209–2.660), with *P* = 0.005 (Table 6). A multivariable logistic regression analysis with adjustment of age, stage, tumor size, and nodal status confirmed a significant association between the luminal A-like subtype with a risk of bone metastasis (OR 1.872, 95% CI 1.044–3.357), with *P* = 0.035.

Liver metastasis was differently distributed among intrinsic breast cancer subtypes ranging from 3.9% (*n* = 11) in TNBC, 5.9% (*n* = 10) in Her2-enriched, 6% (*n* = 29) in luminal A-like subtype, and 6.5% (*n* = 8) in luminal B-like. In addition, luminal subtypes had higher rates of liver metastasis compared to non-luminal subtypes (5.9% vs 4.6%). Using univariable and multivariable binary logistic regression analyses, no specific type of breast cancer was significantly correlated for liver metastasis.

Brain metastasis was identified in 20 patients (1.9%) and was distributed in different rates according to the intrinsic breast cancer subtypes, i.e., 1.2% (*n* = 2) in Her2-enriched, 1.7% (*n* = 2) in luminal B-like, 2.06% (*n* = 10) in luminal A-like, and 2.1% (*n* = 6) in TNBC. The frequency of brain metastasis in luminal and non-luminal subtypes was 2% and 1.8%, respectively. Univariable and multivariable binary logistic regression analyses did not show a significant association of certain breast cancer subtype with brain metastasis.

The Her-2 positive expression did not show direct association with an increased risk of distant metastasis as well as with organ-specific metastasis (Table 6). Our previous results showed that positive axillary lymph nodes were the strongest predictor for distant metastasis. Proportions of positive axillary lymph node were higher in pulmonary metastasis (82.6% vs 71.3%), pleural metastasis (82.4% vs 69.7%), bone metastasis (83.7% vs 71.2%), liver metastasis (75.4% vs 72.6%), and brain metastasis (75% vs 72.7%). Binary logistic regression analysis also revealed the significant association of positive lymph node as a risk factor for pulmonary metastasis (OR 1.938, 95% CI 1.210–3.150), with *P* = 0.006; pleural metastasis (OR 1.886, 95% CI 1.081–3.290), with *P* = 0.025; and bone metastasis (OR 2.093, 95% CI 1.285–3.411), with *P* = 0.003 as shown in Table 6.

## Discussion

In this study, more than half of the cases were diagnosed in advanced stage (stage III) and around one fifth of patients were found with metastatic disease (stage IV). In contrast to our findings, 64% of breast cancer patients in the USA were diagnosed in early stages, and 27% and 6% were found in advanced and metastatic diseases, respectively [20]. In comparison with other Asian countries, 18.7% of breast cancer patients in China were diagnosed in stage III disease while more than half of the patients in India were found in stages III–IV disease [21]. Our study, therefore, indicated that the proportions of breast cancer patients diagnosed in an advanced stage and metastatic disease in Indonesia are relatively higher. We then analyzed some potential factors associated with advanced-stage presentation in our cohort. In Table 2, we showed that lower education levels and residence in a rural area were associated with advanced stage at diagnosis. The delayed breast cancer diagnosis might reflect the socioeconomic disparity in Indonesia as another study suggests that improving education and healthcare access is associated with a gradual reduction of advanced stages at diagnosis [22]. Our previous study found that education levels and lower household expenditure correlated with awareness and cancer screening participation of Indonesian women [23] indicating the potential interaction between cancer awareness and late stages at diagnosis. To some extent, lower socioeconomic status is a risk factor for late presentation and advanced breast cancer stages at diagnosis worldwide and the odds ratio is higher in low-income countries [24–26].

Demographic variables were distributed evenly among intrinsic breast cancer subtypes (Table 1). Diagnosis of breast cancer in women younger than 40 years old correlated with a higher risk of metastasis (Table 3and 4) confirming our previous report of aggressive behavior in younger patients [27, 28]. In relation to distant metastasis, younger age and late menopause were associated with increased risks and the odds ratio was higher in the luminal subtype for women younger than 40 years old and in TNBC for women with menopause older than 50 years old. Abubakar et al. did not find any specific association of age at diagnosis and menopause with recurrence and mortality rates of breast cancer patients [29]. Younger age at diagnosis is correlated with higher distant metastasis [30] although Purushotham et al. showed the inverse correlation [31]. Specific measures might be addressed to younger women with breast cancer as the proportion is relatively high as well as a higher risk of distant metastasis [32]. Patient age has also been incorporated in the treatment plans by considering aggressive therapy, physical functioning, quality of life, and body image [32, 33].

Identification of risk factors associated with distant spread is very crucial in designing breast cancer treatment and surveillance plan after acute treatment. Therefore, identification of determinants associated with an elevated risk of distant metastasis according to breast cancer subtypes as well as some potential interventions to lower the risk has emerged as an important study field in oncology. Distant metastasis involves a complex interaction of primary tumor milieu and systemic factors including cancer cell proliferation, differentiation, angiogenesis, and the microenvironment [34]. Several clinicopathological factors affect specific clinical outcomes of breast cancer management. Tumor size is an established predictor for breast cancer survival rates [35]. The epidemiological studies showed a consistent association between tumor size between 1 and 5 cm with distant metastasis and lymph node infiltration [35, 36]. The most commonly accepted concept of the association between tumor size and metastasis risk is that during cancer progression, cancer cells accumulate specific accessional genetic events resulting in the additional ability to further spread into regional lymph nodes and distant organs [37]. In this study, we found that infiltration to the skin and chest wall (T4a-c) had a higher risk for distant metastasis particularly in the luminal A-like subtype although direct comparison of tumors larger and smaller than 5 cm did not show different risks of metastasis (Table 3 and 4). Although skin infiltration represents the extension of breast cancer, some studies did not show its direct impact on patients’ survival and prognosis [38, 39] rather than accompanied by axillary nodal infiltration [40].

Although in the era of genomic profiling and increased feasibility of incorporating deep sequencing for cancer management [41], the nodal status remains the most important risk factor of survival and metastasis as we also showed in our study. Therefore, node status is also an important determinant in the decision-making for breast cancer treatment. In addition to tumor size and nodal status, the risk of metastasis to a large extent has also been associated with intrinsic breast cancer subtypes. We found positive axillary lymph nodes (N1-3) had a higher risk of distant metastasis, particularly in the luminal A-like breast cancer subtype. In other subtypes, T4 and N1-3 did not significantly correlate with distant metastasis suggesting that small size of the tumor and negative nodal status in non-luminal types also had a high risk of distant spread.

Breast cancer has been viewed as a heterogeneous disease with substantial underlying differences in the molecular alterations and clinical course. However, specific subtypes of breast cancer in our cohort did not show a significant association with the risk of distant metastasis (Table 5). Several studies have shown a higher risk of distant metastasis in Her-2-enriched [42] and TNBC subtype [43] and a lower risk of distant spread in the luminal subtype [42]. In this study, we showed specific risk factors of distant metastasis in luminal A-subtype, i.e., advanced stages (OR 2.160), tumor infiltration to skin and chest wall (OR 2.685), and positive axillary lymph nodes (OR 2.571). In addition, chemotherapy using a combination of anthracycline-taxane showed relative benefits to reduce the risk of distant metastasis in the luminal A-like subtype (OR 0.417) compared to anthracycline-based chemotherapy. Patients with luminal A-like subtype generally have better disease-free and overall survival than other subtypes although in the context of neoadjuvant chemotherapy, it has the lowest rates of pathological complete response [44]. Luminal A-like subtype develops mainly through the estrogen pathway; thus, adjuvant endocrine therapy remains the gold standard for treatment. Recent clinical trials have suggested omitting chemotherapy in node-negative luminal A subtype if the risk is lower [45]. In a high-risk luminal A, however, 30–50% of patients relapse and develop distant metastasis particularly in cases with node-positive tumors and younger patients [46]. Some retrospective studies reported that standard anthracycline-based chemotherapy was not effective in luminal A-subtype [47–49]. However, chemotherapy showed some benefits in high-risk luminal subtypes particularly with positive lymph nodes [47]. Since the majority of our cohort have positive lymph nodes, we revealed specific attributable factors of distant metastasis risks in the luminal A-like subtype. In a meta-analysis involving more than 100,000 breast cancer patients, adding taxanes to anthracycline has been associated with significantly decreased risk of recurrence and cancer-associated death [50] as confirmed by our study. In addition, luminal breast cancer patients who effectively received chemotherapy and continued to receive effective treatment after distant metastasis were reported to have longer survival [51].

The four intrinsic breast cancer subtypes show different predilections for organ-specific metastasis. In our study, non-luminal breast cancer subtypes and positive node had a higher risk of pulmonary metastasis (OR 1.445 and 1.938, respectively). In accordance with our study, distant spread to the lung has been found more frequently in non-luminal triple-negative breast cancer [42, 52, 53]. The bone was found as the predilection of metastasis in the luminal A subtype (OR 1.601) that supported previous reports [54, 55]. The most common sites for distant metastasis from breast cancer are the bone (65%), followed by the liver, lung, and brain [56]. Distant spread to certain organs is an orderly process known as metastatic organotropism and is regulated by a number of factors including intrinsic breast cancer subtypes, metabolic changes, molecular alterations of the cancer cells, host immune responses, and tumor microenvironment [34, 57]. Coordinated activation of several pathways including Notch, Wnt/ß-catenin, and Hedgehog, as well as proteins including COX2, metalloproteinases, and vascular endothelial growth factor (VEGF) collectively facilitate the release of cancer cells and the infiltration into distant organs [53]. In terms of organ-specific metastasis, growth factors, interleukin, RANKL, and Scr pathways are activated in luminal breast cancer to mediate bone metastasis [57]. Chemokines, interleukin, HIF, and Wnt signaling are activated in Her2-enriched and luminal breast cancer subtypes to mediate liver metastasis [57]. In addition, growth factors, tumor growth factor-beta (TGFβ), and COX2 are responsible for lung metastasis in non-luminal breast cancer subtypes [34]. Future studies to understand more details of the association of molecular subtypes and organ-specific metastasis will improve the future clinical management, mode of surveillance, and new targeted treatment for breast cancer patients.

Distant metastasis has long been associated as the main cause of mortality in breast cancer. Although breast cancer has emerged as a significant health burden in Indonesia due to the increasing incidence and large proportion of advanced stages at presentation, no previous study has evaluated the attributable clinicopathological risks of distant spread. Research that evaluates current clinical practice and surveillance of breast cancer in our population is also limited. However, our study had some limitations associated with the naturally retrospective case-control design and shorter follow-up. The analysis of dynamic transition into a metastatic state, direct evaluation of treatment effects, and contribution of comorbidities could also not be performed. Larger studies involving multiple centers or population-based studies with longer follow-up are required to confirm our study.

## Conclusion

We identified tumor infiltration to the skin and chest wall and positive axillary lymph nodes as risk factors of distant metastasis in breast cancer. Certain intrinsic subtypes of breast cancer have different patterns and tropisms for organ-specific distant metastasis. In addition, we found a significant association of lower education levels and residence in a rural area with more advanced stages of breast cancer diagnosis in Indonesia. Improving clinical management and surveillance plans are warranted for patients with a higher risk of distant metastasis. In addition, public health interventions and health system improvements to reduce breast cancer diagnosis in advanced stages are also required.

## Data Availability

The dataset is available upon reasonable request to the corresponding author.

## Abbreviations

AJCC: American Joint Committee on Cancer
BMI: Body mass index
BSA: Body surface area
ER: Estrogen receptor
Her2: Human epidermal growth factor receptor-2
LMIC: Low- and middle-income countries
mSBR: Modified Scarff-Bloom and Richardson system
OR: Odds ratio
PR: Progesterone receptor
SD: Standard deviation
SE: Standard error
TNM: Tumor node metastasis
TNBC: Triple-negative breast cancer
WHO: World Health Organization
